# Time-dependent effect of intense capsule-coffee and bleaching on the color of resin-infiltrated enamel white spot lesions: an *in vitro* study

**DOI:** 10.7717/peerj.14135

**Published:** 2022-10-06

**Authors:** Hanin E. Yeslam, Saeed J. AlZahrani

**Affiliations:** Department of Restorative Dentistry, King Abdulaziz University, Jeddah, Saudi Arabia

**Keywords:** Resin infiltration, Incipient caries lesions, White spot lesions, Esthetic treatment, Capsule coffee, Conservative dentistry, Restorative dentistry, ICON, Dental caries, Bleaching

## Abstract

**Background and Objectives:**

White spot lesions (WSLs) are a common problem that can be conservatively managed by resin infiltration. Consumption of dark beverages such as coffee causes staining of dental hard tissues, which can deteriorate the esthetic qualities of treated WSLs. The aims of this study were to investigate the effect of dark coffee heavy consumption on ICON^®^ resin infiltrated WSLs and the influence of bleaching on them after staining.

**Methods:**

Twenty surfaces from sound human extracted third molars were used in the study. Two square-shaped buccal and lingual window areas had artificially created WSLs (received buccal resin infiltration afterward). Using VITA Easyshade, the baseline Δ*E* was recorded. Espresso coffee was used to immerse all surfaces for 8 days. The color coordinates according to CIE LAB were recorded for all surfaces at 2, 4, 6, and 8 days of immersion, and Δ*E* was calculated at each timepoint. After 8 days, in-office bleaching was applied to all surfaces according to the manufacturer’s instructions and the color. Coordinates and Δ*E* were recorded. For statistical analysis, an independent sample *t*-test was used to compare each group. A general linear mixed model (GLMM) repeated measure ANOVA was applied for statistical analysis of L^*^and changes due to staining over time.

**Results:**

Analysis of time as a main effect on the L values of surfaces was statistically highly significant (*p* < 0.01). The interaction of time with surfaces under investigation and type of surface (test vs. control) as a main effect were non-significant (*p* = 0.47 and *p* = 0.35, respectively). Bleaching showed a significant difference in color lightness in both test and control surfaces compared to the mean *L* value at 8 days of staining (*p* < 0.01).

**Conclusion:**

Capsule-coffee consumption gradually influences the esthetic of resin infiltration on treated teeth. However, bleaching materials might restore an esthetic shade.

## Introduction

White spot lesions (WSL) are non-cavitated incipient caries of enamel lesions. It is a common problem encountered in patients following treatment with orthodontic appliances. This is caused by the accumulation of plaque around the braces and band combined with the diminished ability of patients to maintain proper oral hygiene applications (Mizrahi, 1982). Enamel white lesion appears after calcium and phosphate ions are leached out due to exposure to organic acid produced by cariogenic bacteria, which leaves internal porosities that affect the refractive index of the enamel surface (Kidd & Fejerskov, 2004).

A meta-analysis study showed that the incidence of WSL in patients during fixed orthodontic appliance treatment was about 45.8%, while its prevalence in patients undergoing orthodontic treatment was 68.4% (Sundararaj et al., 2015). Resin infiltration is an intermediary management option between preventive and restorative treatment modalities for managing WSLs (Hammad, El-Wassefy & Alsayed, 2020). Resin infiltration is based on the capillary infiltration of light-cured low-viscosity tri-ethylene glycol di-methacrylate (TEGDMA) resin into the lesion to inhibit its progression by blocking the pores on the surface of the demineralized enamel (Mueller et al., 2006; Paris & Meyer-Lueckel, 2010). However, TEGDMA-containing resins (including ICON^®^ infiltration resin) are susceptible to staining, posing an esthetic problem when treating WSLs (Araujo et al., 2015; Rey et al., 2014).

Coffee is amongst the most popular beverages consumed worldwide. The convenience of coffee capsules and automated machines have facilitated access to a wide variety of coffee types (Lolli et al., 2020). Studies that report the beneficial health effects of moderate coffee consumption further increased the beverage’s popularity (Grosso et al., 2017; Chrysant, 2017). Unfortunately, consuming coffee and other colored drinks is linked to discoloration of dental hard tissues, infiltration resins, and restorative dental materials (Aswani et al., 2019). However, literature shows conflicting reports on the extent of discoloration in restored dental structures and resin-infiltrated WSLs (Araujo et al., 2015; Gross & Moser, 1977; Zimmerli et al., 2012; Manno et al., 2018). At-home bleaching using peroxide compounds showed promise in improving the color of stained sound and resin-infiltrated tooth structure (Araujo et al., 2015; Bazzi et al., 2012; Jansen et al., 2021). A single application session of 35% hydrogen peroxide bleaching gel to enamel provided some degree of shade change (de Silva Gottardi, Brackett & Haywood, 2006). However, literature shows that two to three applications of in-office bleaching products usually produce favorable results (Mourouzis, Koulaouzidou & Helvatjoglu-Antoniades, 2013; Hubbezoglu et al., 2008; Yami et al., 2020). In 2019, Torres et al. (2019) reported that 35% hydrogen peroxide on bovine enamel successfully increased the lightness of stain-affected resin-infiltrated WSLs, but caused some degree of deterioration in the infiltrated-enamel translucency. On the other hand, Rocha et al. (2020) reported the limited effectiveness of bleaching on resin-infiltrated WSLs affected by darker stains.

There are limited reports in the literature regarding the time-dependent color changes in human enamel and resin-infiltrated WSLs caused by intense exposure to readily available dark-capsule coffee and the effect of bleaching on these surfaces. Thus, this study aimed to investigate the effect of intense exposure to popular dark beverages on the color stability of dental hard tissues treated with the ICON^®^ resin infiltration system. Additionally, to investigate the effect of in-office bleaching on the stained hard tissues.

The null hypotheses of the study were that there is no color change difference between different staining solution exposure times and between infiltrated and non-infiltrated WSLs regardless of staining solution exposure time. Additionally, there is no significant difference regarding the effect of bleaching between stained infiltrated and non-infiltrated WSLs.

## Materials & Methods

The study was exempted by the Institutional Review Board of the Faculty of Dentistry at King Abdulaziz University (proposal number 267-08-21).

### Sample preparation

A total of 20 surfaces from ten extracted sound human third molars were included in this study. All teeth were sterilized according to the guidelines proposed by the Center for Disease Control and Prevention (CDC) (Kohn et al., 2003). Teeth were cleaned and inspected for defects and visible stains that would interfere with the study procedures. The root section of each tooth was embedded in a cylindrical polyvinyl silicon base (Speedex Putty; Coltene Whaledent, Altstätten, Switzerland) for handling purposes. The crown part of the embedded teeth was covered with an acid-resistant nail varnish except for two small (4 × 4 mm) square windows—one on the buccal surface (test (*n* = 10)) and the other on the lingual surface (control (*n* = 10)). All teeth were stored in deionized water at 37 °C.

### Subsurface lesion formation

The coronal portions of the embedded teeth were immersed in a demineralization solution {(2.2 mM CaCl_2_, 10 mM NaH_2_PO_4_, 50 mM acetic acid, 100 mM NaCl, 1 ppm NaF, 0.02% NaN_3_ ; pH 4.5) (Bakhsh et al., 2018; Bakry et al., 2018), 16 mL demineralization solution per exposed enamel surface (32 mL_solution_/tooth)} for 6 days. The total volume of the demineralization solution used was determined using the following formula:

\begin{eqnarray*}{\mathbf{V olume}}_{\mathbf{total}}=2{\mathrm{mL}}_{\mathrm{solution}}/1~{\mathrm{mm}}_{\mathrm{enamel~ area}}^{2}\text{(Ferreira et al, 2007)}. \end{eqnarray*}

**Figure 1:**
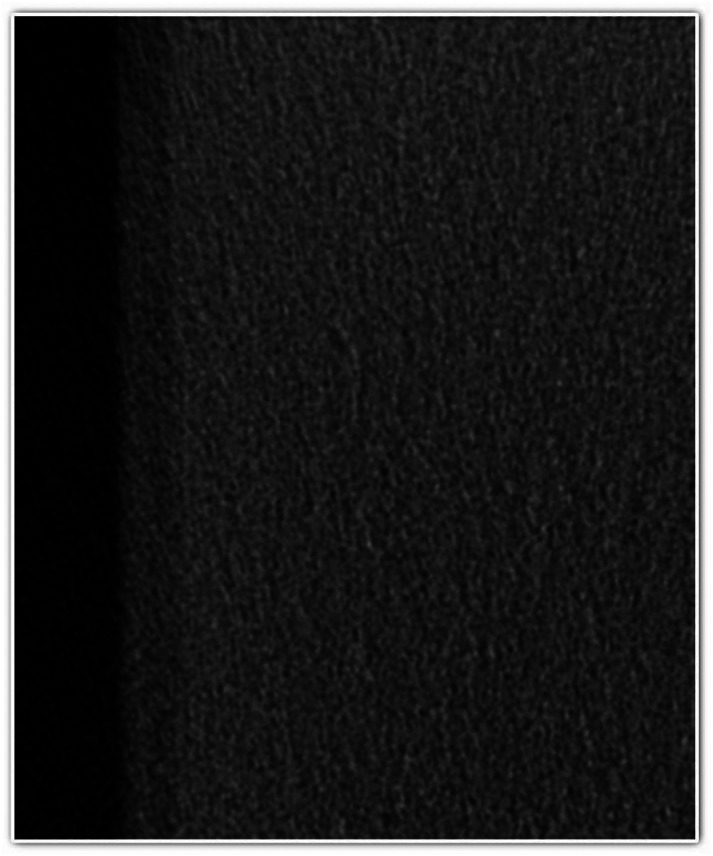
A micro-CT scan picture of a white spot lesion (WSL) that was created on an exposed enamel surface.

Using this volume of similar demineralization solutions results in an almost 43 µm deep subsurface lesion (Magalhães et al., 2009). The establishment of sub-enamel demineralized lesions on each tooth’s exposed buccal and lingual surfaces was validated using micro-computed tomography (SkyScan 1172; Kontich, Belgium) (Oliveira et al., 2020). Figure 1 shows a micro-CT image of the artificial WSLs created.

### Resin infiltration

Infiltration resin (ICON; DMG, Hamburg, Germany) was applied to the enamel surface of the buccal windows of teeth following the manufacturer’s instructions. After drying the teeth, Icon-Etch was applied for 2 min and then suctioned off and rinsed with deionized water for 30 s. Subsequently, the buccal surfaces were rinsed with 100% ethanol for 30 s and then air-dried. Icon-resin infiltration gel was applied after that for 3 min using a smooth surface ICON applicator with continuous massaging. The infiltrated buccal surfaces were then cured using an LED curing unit (3M Elipar™ LED curing light; 3M ESPE, Saint Paul, MN, USA) for 40 s with 1,200 mW/cm^2^ irradiance. A second layer of ICON-infiltration resin was applied and light cured. The exposed lingual enamel surfaces of teeth were not infiltrated and served as a positive control. All teeth were then stored in deionized water at 37 °C for 24 h; then, baseline color measurements were recorded.

### Baseline color measurement

Color measurement was performed against a white background under the same light source (daylight LED bulb) during all stages of the study and by one investigator (H.Y.) to avoid variability in color measurements. The tip of the spectrophotometer was positioned vertically, in a marked location, resting on the surface to be measured. To standardize color measurement throughout the study, an average of three readings on each surface were recorded for each color parameter.

Baseline spectrophotometric color measurements were determined for the test (buccal), and control (lingual) surfaces using the CIE (Commission International de L’Eclairage; Luo, 2014) L*a*b* parameters using a handheld spectrophotometer (VITA Easy shade advance, VITA Zahnfabrik, Bad Säckingen, Germany), where L* indicates lightness (a value of 100 indicates white and zero indicates black); a* represent red (positive) and green colors (negative); b* represents yellow (positive) and blue colors (negative). ${L}_{v}^{\ast }$ was recorded for test and control surfaces. L_v_ represents the tooth structure’s lightness difference in color space between the measured tooth shade and the closest matching VITA classical (A1-D4 shades), where higher values are positive and lower values are negative.

### Coffee immersion procedures and color change

Following resin infiltration, all the samples were immersed in capsule-coffee staining solution (Starbucks Espresso Roast coffee capsules by Nespresso, Nestlé S.A., Vaud, Switzerland) for a total of 8 days. In a study by Ertas et al. (2006), it was reported that an average of 3.2 cups of coffee is consumed daily by average coffee drinkers and the average duration per cup was around 15 min (Ertas et al., 2006). Therefore, 24 h of storage in coffee would correspond to almost one month of coffee drinking (Ertas et al., 2006; Gamal, Safwat & Abdou, 2022; Hasanain, 2022). Consequently, eight days of 24 hours/day storage would correspond to 8 months of coffee drinking. The coffee was prepared using the espresso brewing setting in a 19-bar high-pressure pump coffee capsule machine (Nespresso lattissima touch; Nespresso by De’Longhi, Treviso, Italy). The coffee solution was allowed to cool down to 37 °C before immersing the teeth. The immersed teeth were kept in the coffee solution and stored in an incubator at 37 °C (Memmert, Schwabach, Germany). The coffee staining solution was replaced daily with a freshly brewed coffee solution.

After every two days of immersion in the staining solution, the samples were removed and rinsed gently with deionized water and dried with absorbent paper. The color of exposed surfaces (both test and control surfaces) was recorded using the CIE L*a*b* parameters in the spectrophotometer (VITA Easy shade advance; VITA Zahnfabrik, Bad Säckingen, Germany), as described above in the Baseline Color Measurement Section.

The color change due to immersion (ΔE_x_) in the capsule-coffee staining solution was determined using the CIE 1976 L* a* b* (Luo, 2014) color system according to the following formula:

\begin{eqnarray*}\Delta {\mathbf{E}}_{\mathbi{x}}& & =\sqrt{{ \left( {\mathbi{L}}_{\mathbi{staining}(\mathbi{xdays})}-{\mathbi{L}}_{\mathbi{baseline}} \right) }^{2}+{ \left( {\mathbi{a}}_{\mathbi{staining}(\mathbi{xdays})}-{\mathbi{a}}_{\mathbi{baseline}} \right) }^{2}+{ \left( {\mathbi{b}}_{\mathbi{staining}(\mathbi{xdays})}-{\mathbi{b}}_{\mathbi{baseline}} \right) }^{2}} \end{eqnarray*}

\begin{eqnarray*}& & =\sqrt{\Delta {\mathbi{L}}_{\mathbi{x}}^{2}+\Delta {\mathbi{a}}_{\mathbi{x}}^{2}+\Delta {\mathbi{b}}_{\mathbi{x}}^{2}} \end{eqnarray*}

where _x_ indicates the number of days of immersion in the staining solution. ΔE_2_, ΔE_4_, ΔE_6_, and ΔE_8_ were determined as representing color changes after 2, 4, 6, and 8 days of immersion, respectively. Average ΔE_*x*_ values from the test and control surfaces were recorded at each time point.

Change in lightness ${L}_{v}^{\ast }$ due to capsule-coffee immersion (ΔL${}_{\mathrm{vx}}^{\mathrm{\ast }}$) was determined according to the following formula:

\begin{eqnarray*}\Delta {\mathbi{L}}_{\mathbi{vx}}={\mathbi{L}}_{\mathbi{v~ staining}(\mathbf{x~ days})}-{\mathbi{L}}_{\mathbi{v~ baseline}} \end{eqnarray*}

where _x_ indicates the number of days of immersion in the capsule-coffee staining solution. ΔL${}_{v2}^{\ast }$, ΔL${}_{v4}^{\ast }$, ΔL${}_{v6}^{\ast }$, and ΔL${}_{v8}^{\ast }$ were determined and represent color changes after 2, 4, 6, and 8 days of immersion, respectively.

### Bleaching and color change

After 8 days of staining, teeth were rinsed with deionized water and dried using air jets. An approximately one mm thick layer of 35% hydrogen peroxide in-office bleaching gel (Whiteness HP Blue; FGM, Joinville, Brazil) was applied to both test and control surfaces following the manufacturer’s instructions. The gel was allowed to remain in contact with the enamel surface of teeth for 40 min, after which the gel was suctioned off, and the enamel surfaces were rinsed thoroughly with deionized water.

Color change due to bleaching was calculated using the following formula:

\begin{eqnarray*}\Delta {E}_{\mathrm{bx}}& & =\sqrt{{ \left( {\mathbi{L}}_{\mathbi{bx}}-{\mathbi{L}}_{\mathbi{staining}(8\mathbi{days})} \right) }^{2}+{ \left( {\mathbi{a}}_{\mathbi{bx}}-{\mathbi{a}}_{\mathbi{staining}(8\mathbi{days})} \right) }^{2}+{ \left( {\mathbi{b}}_{\mathbi{bx}}-{\mathbi{b}}_{\mathbi{staining}(8\mathbi{days})} \right) }^{2}}& & =\sqrt{\Delta {\mathbi{L}}_{\mathbi{bx}}^{2}+\Delta {\mathbi{a}}_{\mathbi{bx}}^{2}+{\mathbi{b}}_{\mathbi{bx}}^{2}} \end{eqnarray*}

where b indicates post bleaching color measurements, and x indicates the bleaching round. The CIE L*a*b* parameters and ${L}_{v}^{\ast }$ at 8 days of staining were used as the baseline measurement to determine color change after bleaching. Mean ${L}_{vb1}^{\ast }$ and ΔE_b1_ values for the test and control surfaces were recorded. Teeth were stored in deionized water at 37 °C. Both test and control surfaces received a second round of bleaching using 35% hydrogen peroxide in-office bleaching gel (Whiteness HP Blue, FGM, Joinville, Brazil), as described above. CIE L*a*b* color parameters, ${L}_{vb2}^{\ast }$, and Δ*E*_b2_, were recorded.

Change in lightness ${L}_{vb}^{\ast }$ due to bleaching (ΔL${}_{\mathrm{vbx}}^{\ast }$) was determined according to the following formula:

\begin{eqnarray*}\Delta {\mathbi{L}}_{\mathbi{vbx}}={\mathbi{L}}_{\mathbf{v}\mathbi{bx}}-{\mathbi{L}}_{\mathbi{v~ staining}(8\mathbi{days})} \end{eqnarray*}

\begin{eqnarray*}\Delta \Delta {\mathbi{L}}_{\mathbi{vb}}={\mathbi{L}}_{\mathbf{v}\mathbi{b}2}-{\mathbi{L}}_{\mathbi{vb}1} \end{eqnarray*}

where _b_ indicates post-bleaching color measurements and _x_ indicates the number of bleaching rounds.

Mean values of ΔL_*vbx*_^∗^, Δ Δ***L***_***vb***_^∗^, and Δ*E*_bx_ were statistically analyzed.

Change in the change of color (ΔΔE_*b*_) between first and second bleaching rounds was determined according to the following formulas:

\begin{eqnarray*}\Delta \Delta {\mathbi{E}}_{\mathbi{b}}=\Delta {\mathbi{E}}_{\mathbi{b}2}-\Delta {\mathbi{E}}_{\mathbi{b}1} \end{eqnarray*}

where _b_ indicates post-bleaching color measurements and _x_ indicates the number of bleaching rounds. Mean Δ ΔE_b_ of test and control surfaces were statistically analyzed.

### Statistical analysis

The color parameter ΔL${}_{v}^{\mathrm{\ast }}$ and color change ΔE were statistically analyzed. All the color-related data were collected, tabulated, and analyzed statistically. Statistical analysis was performed using SPSS (version 20). Data organization and graphical representation were conducted using Microsoft Office Excel. The following quantitative variables were described; mean, standard deviation (SD), range (minimum–maximum values), standard error (SE), 95% confidence interval of the mean, and coefficient of variation. A Shapiro–Wilk normality test was used to test all quantitative variables for hypothesis normality to choose the appropriate parametric and non-parametric tests. All tested variables were normally distributed; therefore, parametric tests were used. A general linear mixed model (GLMM) repeated measure ANOVA was conducted for statistical analysis of ΔL ${}_{v}^{\ast }$ and Δ*E* changes due to staining over time and changes due to bleaching rounds with a significance level of *p* <  0.05 (S), while *p* < 0.001 was considered a highly significant (HS) difference. Multiple pairwise comparisons were conducted using the Bonferroni method. Two-tailed tests were utilized for the analysis of all statistical tests. Independent sample *t*-test was used for the comparison between every two groups.

## Results

A Shapiro–Wilk test of normality for all the variables showed a normal distribution of the measured results, and therefore, parametric tests were used for comparing every two groups.

### Assessment of staining effect on enamel surface lightness

Table 1 shows the ${L}_{v}^{\ast }$ color lightness parameter mean values and standard deviation (SD) of the test (received ICON infiltration treatment) and control (no ICON infiltration done) surfaces at baseline and after 2, 4, 6, and 8 days of immersion in the capsule-coffee staining solution.

**Figure 2:**
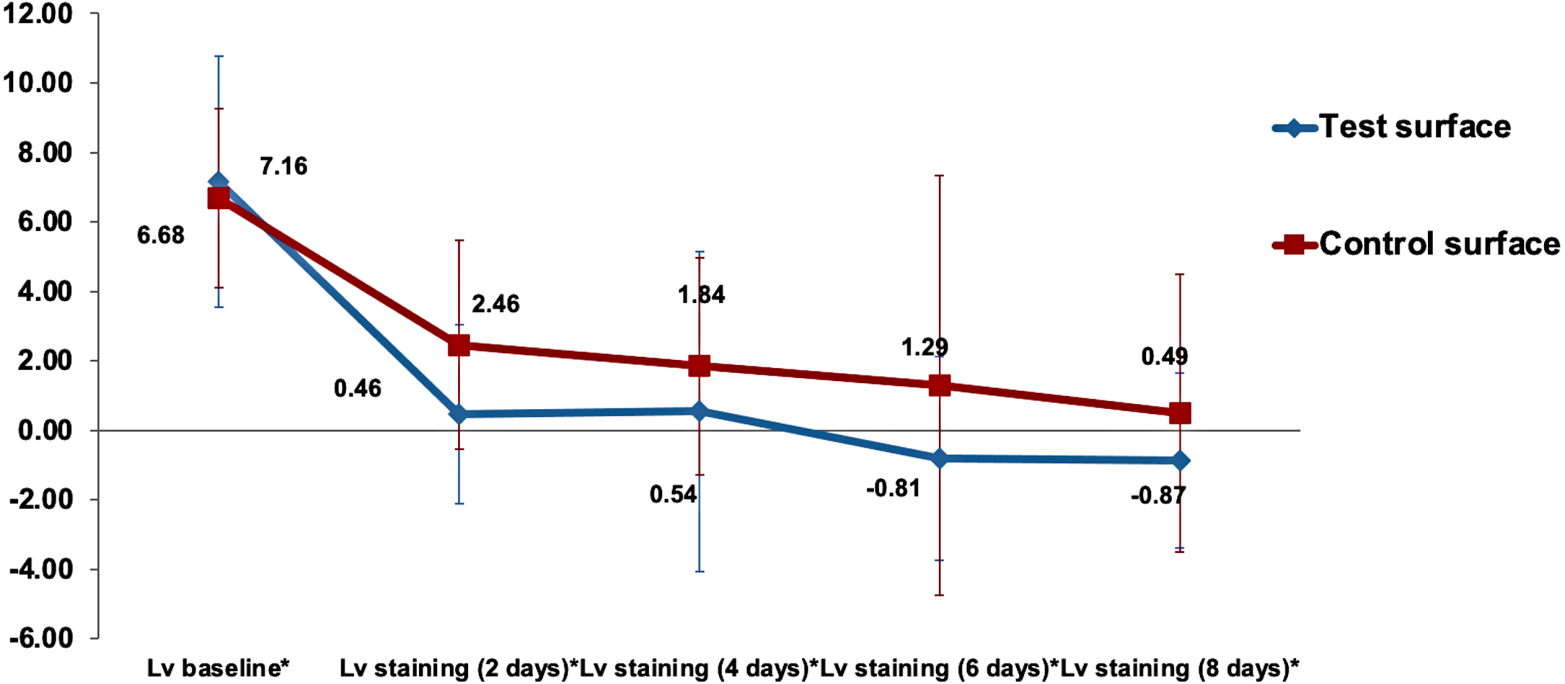
The gradual decrease in the mean value of ${L}_{vx}^{\ast }$ color parameter for both test and control surfaces over time. ${L}_{vx}^{\ast }$ is the lightness difference in color space between the measured tooth shade at staining time point *x* and the closest matching VITA classical (A1-D4 shades).

The gradual decrease in the mean values of the ${L}_{v}^{\ast }$ color parameter over time for both test and control enamel surfaces in the study is demonstrated in Fig. 2. Analysis of time as a main effect on the ${L}_{v}^{\ast }$ values of surfaces was statistically highly significant (*p* < 0.001). Both the interaction of time with surfaces under investigation and the type of surface (test vs. control) as a main effect were non-significant (*p* = 0.47 and *p* = 0.35, respectively).

**Table 1:** This table details the mean L value for the test and control surfaces at baseline and during the staining procedure. ${}^{\ast }{L}_{v}^{\ast }$ is lightness difference in color space between the measured tooth shade and the closest matching VITA classical.

**L** ^*^	**Surface Group**	**Mean**	**SD** ^a^	**N**
baseline	Test	7.16	3.62	10
	Control	6.68	2.58	10
L_*staining*(2*days*)_	Test	0.46	2.59	10
	Control	2.46	3	10
L_*staining*(4*days*)_	Test	0.54	4.61	10
	Control	1.84	3.13	10
L_*staining*(6*days*)_	Test	−0.81	2.93	10
	Control	1.29	6.04	10
L_*staining*(8*days*)_	Test	−0.87	2.51	10
	Control	0.49	4.012	10

**Notes.**

* L is lightness color parameter.

a SD is standard deviation.

Multiple comparisons of the changes in surface lightness (ΔL${}_{v}^{\ast }$) between the various staining times regardless of the surface type by the Bonferroni method showed highly significant changes when (${L}_{v}^{\ast }$) was compared to baseline values (*p* < 0.001) and statistically significant differences between 2 days staining and the 8 days staining (*p* = 0.028). Even though there was a slight decrease in the surface lightness in the test group over the control group, as shown in Fig. 2. However, no statistically significant difference between the surface lightness of test and control surfaces was detected using the Bonferroni method (*p* = 0.35).

**Figure 3:**
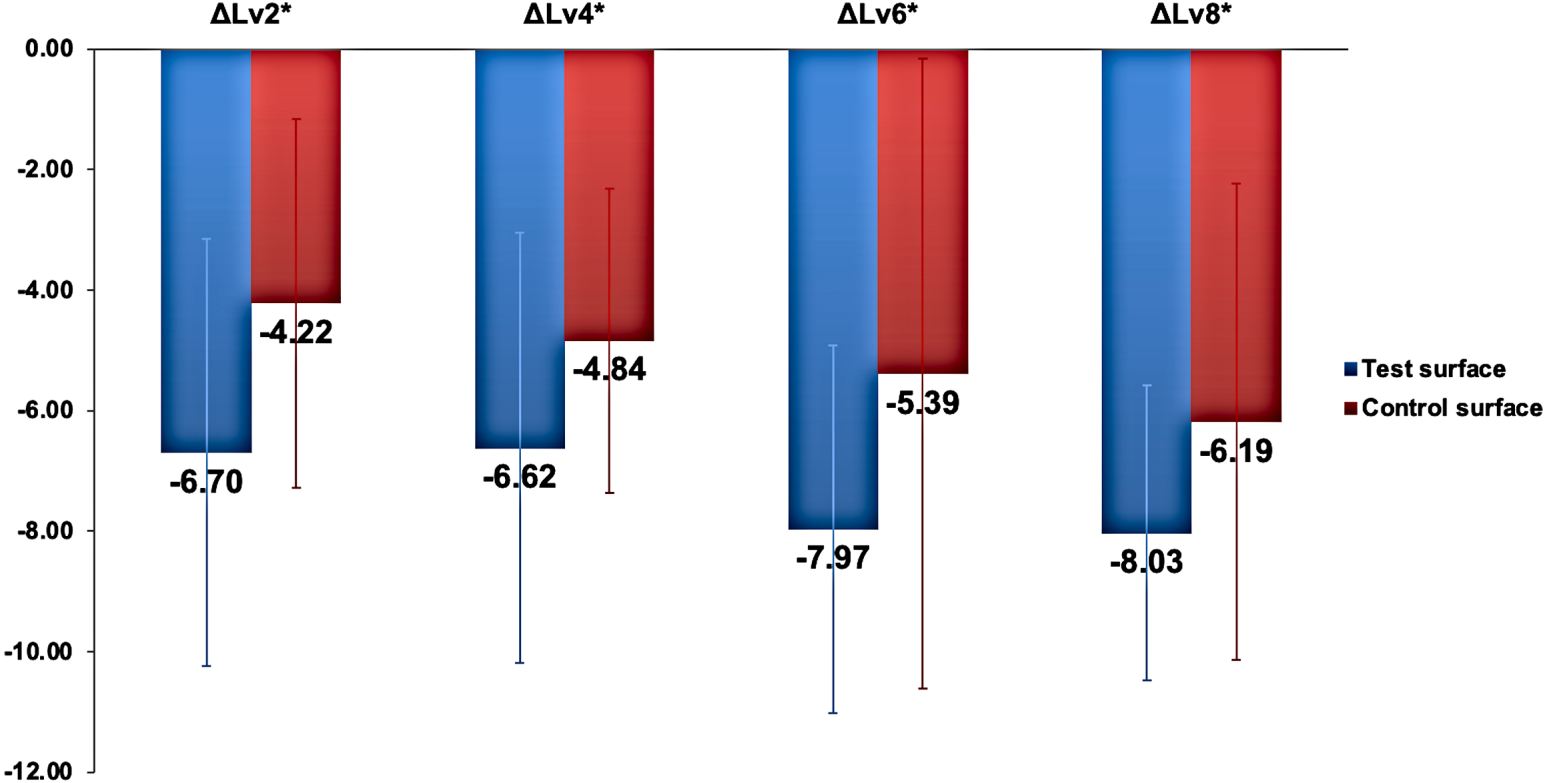
The changes in lightness ($\Delta {L}_{vx}^{\ast }$) in test and control surfaces due to capsule-coffee staining at different time points. Δ*L*_*vx*_ * represents *L*_*v*_ staining(x days) ∗_*Lv*_ baselin *e*^∗^ , where *x* represents the number of staining days.

There were no statistically significant differences (*p* >0.05) in the change in lightnes*s* (ΔL_*v* *staining*(*xdays*)_) relative to lightness at baseline (ΔL_*v*.*baseline*_) between the two surfaces (ΔL_*vx*_) when analyzed using the independent sample *t*-test at all time points. The changes in lightness at different time points relative to baseline *L*_*v*_ values are presented in Fig. 3.

### Assessment of staining effect on enamel surface change of color

Table 2 shows the change of color (ΔE_*x*_) mean values and standard deviation (SD) of the test (received ICON infiltration treatment) and control (no ICON infiltration done) surfaces after 2, 4, 6, and 8 days of immersion in the capsule-coffee staining solution.

**Table 2:** Mean change of color ΔE_x_ values for the test and control surfaces at different time points of the staining procedure.

ΔE_x_^*^	**Surface Group**	**Mean**	**SD** ^a^	**N**
ΔE_2_	Test	**9.58**	**1.37**	10
	Control	**5.03**	**2.27**	10
ΔE_4_	Test	**9.44**	**1.82**	10
	Control	**5.11**	**1.79**	10
ΔE_6_	Test	**10.26**	**1.69**	10
	Control	**5.43**	**2.33**	10
ΔE_8_	Test	**10.86**	**1.16**	10
	Control	**5.89**	**2.47**	10

**Notes.**

* Δ*E*_x_ is change of color after x days of staining.

a SD is standard deviation.

**Figure 4:**
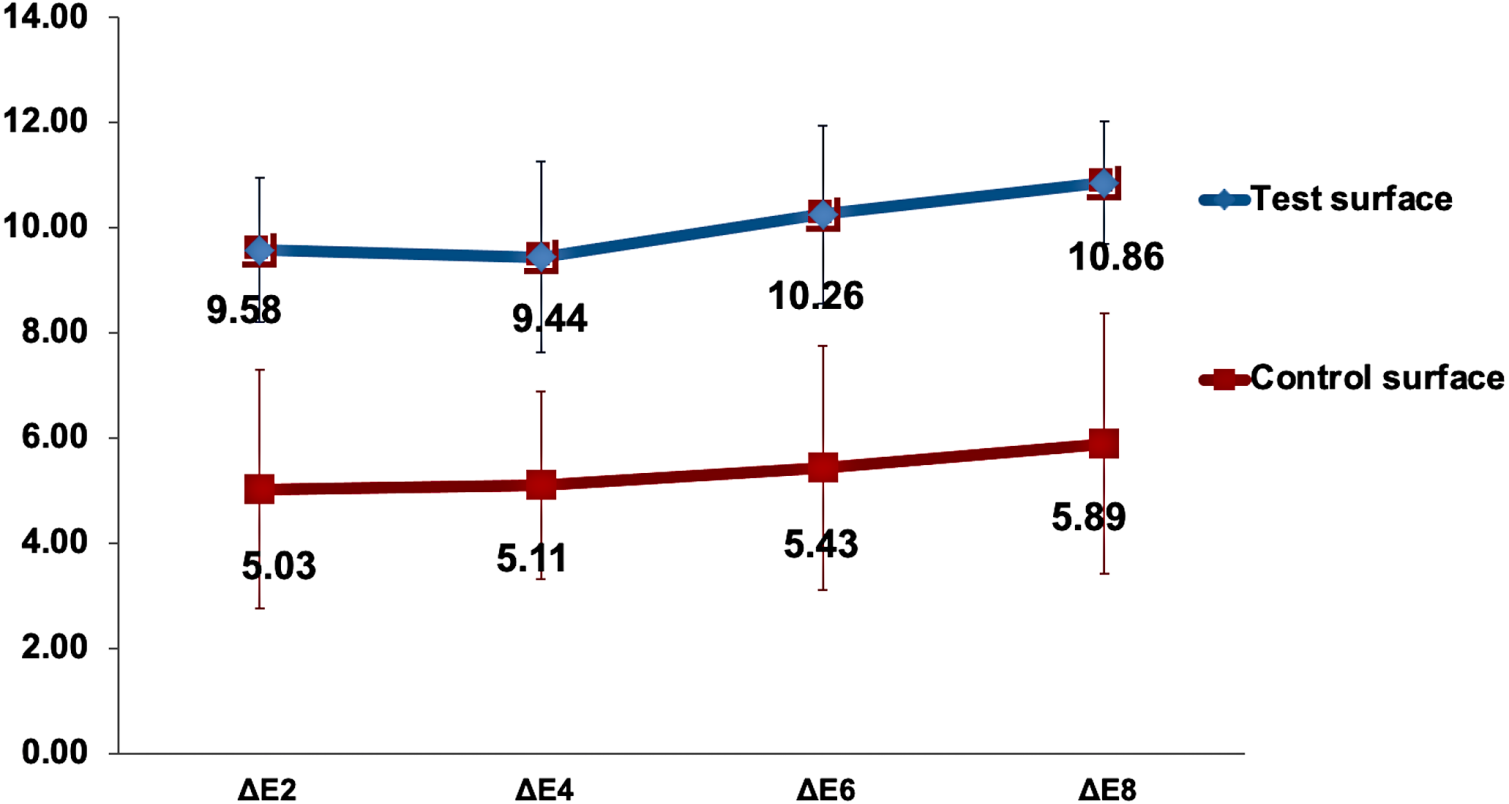
Changes in the mean Δ*E*_*x*_ value for test and control surfaces over time. ΔE_*x*_ represents the change in color of test and control surfaces between *x* days of staining and baseline.

The changes in the mean (ΔE_*x*_) values over time for both test and control enamel surfaces in the study are demonstrated in Fig. 4. Mauchly’s test of sphericity was non-significant, indicating non-violation of the sphericity assumption. Analysis of time as a main effect on the (ΔE_*x*_) values of surfaces was statistically highly significant (*p* < 0.001). In contrast, time interaction with surfaces under investigation was non-significant (*p* = 0.7). However, the color change (ΔE_*x*_) when analyzing the type of surface as a main effect was statistically highly significantly higher in the test surfaces than in the control surfaces (*p* <  0.001). Pairwise comparison between test and control surfaces using the Bonferroni method revealed a highly significant difference between the surfaces in response to the staining solution (*p* < 0.001).

Multiple comparisons of color change between the time points of immersion in the capsule-coffee solution, regardless of the surface type, were determined with the Bonferroni correction method at a significance level of *p* < 0.05. There was a statistically significant increase in ΔE_*x*_ between the eighth and second days and between the fourth and eighth days of staining (*p* = 0.03 and *p* = 0.01, respectively).

### Assessment of bleaching effect on lightness of capsule-coffee-stained enamel surfaces

Table 3 shows the mean lightness and change of color values for the test and control surfaces with each bleaching round.

**Table 3:** Mean lightness and change of color values for the test and control surfaces with bleaching.

**Color parameter**	**Surface Group**	**Mean**	**SD** ^a^	**N**
*L*_v staining (8 days)_ ^*^	*Test*	*−0.87*	*2.51*	*10*
	*Control*	*0.49*	*4.01*	*10*
*L*_*vb*1_ ^*^	*Test*	*4.72*	*2.94*	*10*
	*Control*	*5.61*	*2.54*	*10*
*L*_*vb*2_ ^*^	*Test*	*6.51*	*2.49*	*10*
	*Control*	*7.20*	*2.05*	*10*
Δ*E*_b1_	*Test*	*6.63*	*1.53*	*10*
	*Control*	*4.58*	*2.08*	*10*
Δ*E*_b2_	*Test*	*8.74*	*1.35*	*10*
	*Control*	*6.29*	*2.51*	*10*

**Notes.**

^∗^*L*_*vbx*_∗ is the lightness and ΔE _bx_ is change of color due to bleaching, where _x_ is the bleaching round.

a SD is standard deviation.

**Figure 5:**
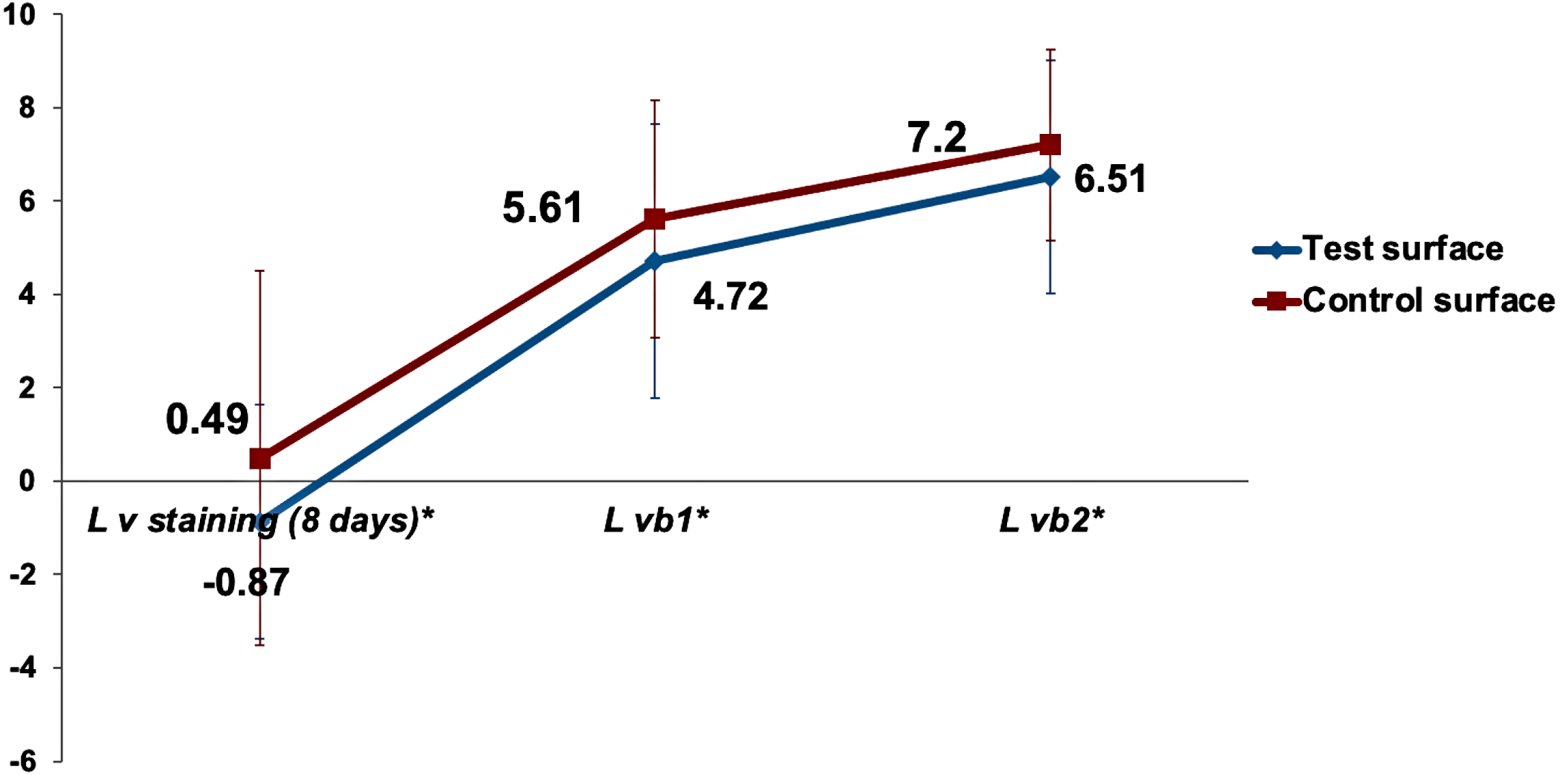
The gradual increase in the mean values of the ${L}_{vbx}^{\ast }$ color parameter due to bleaching for test and control surfaces. ${L}_{vbx}^{\ast }$ is the lightness difference in color space between the measured tooth shade after bleaching round x and the closest matching VITA classical (A1-D4 shades) *L*_*v*_ staining (8 days)^∗^ is the lightness difference in color space between the measured tooth shade after 8days of staining and the closest matching VITA classical (A1-D4 shades).

General linear model repeated measure mixed design ANOVA was used to analyze the effect of bleaching rounds on the lightness of test and control surfaces. The gradual increase in the mean values of the ${L}_{vbx}^{\ast }$ color parameter with bleaching round for both test and control enamel surfaces is demonstrated in Fig. 5. Mauchly’s test of sphericity was significant, indicating a violation of sphericity assumption. Therefore, Greenhouse-Geisser correction was applied. Bleaching round number as a main effect on the color parameter ${L}_{v}^{\ast }$ was statistically highly significant (*p* < 0.001). The mean color parameter ${L}_{vb2}^{\ast }$ was significantly higher compared to the mean ${L}_{v~staining(8days)}^{\ast }$ value and the mean ${L}_{vb1}^{\ast }$ value in both test and control surfaces (*p* < 0.001). In contrast, the interaction of bleaching round and type of surfaces was statistically non-significant (*p* = 0.69).

**Figure 6:**
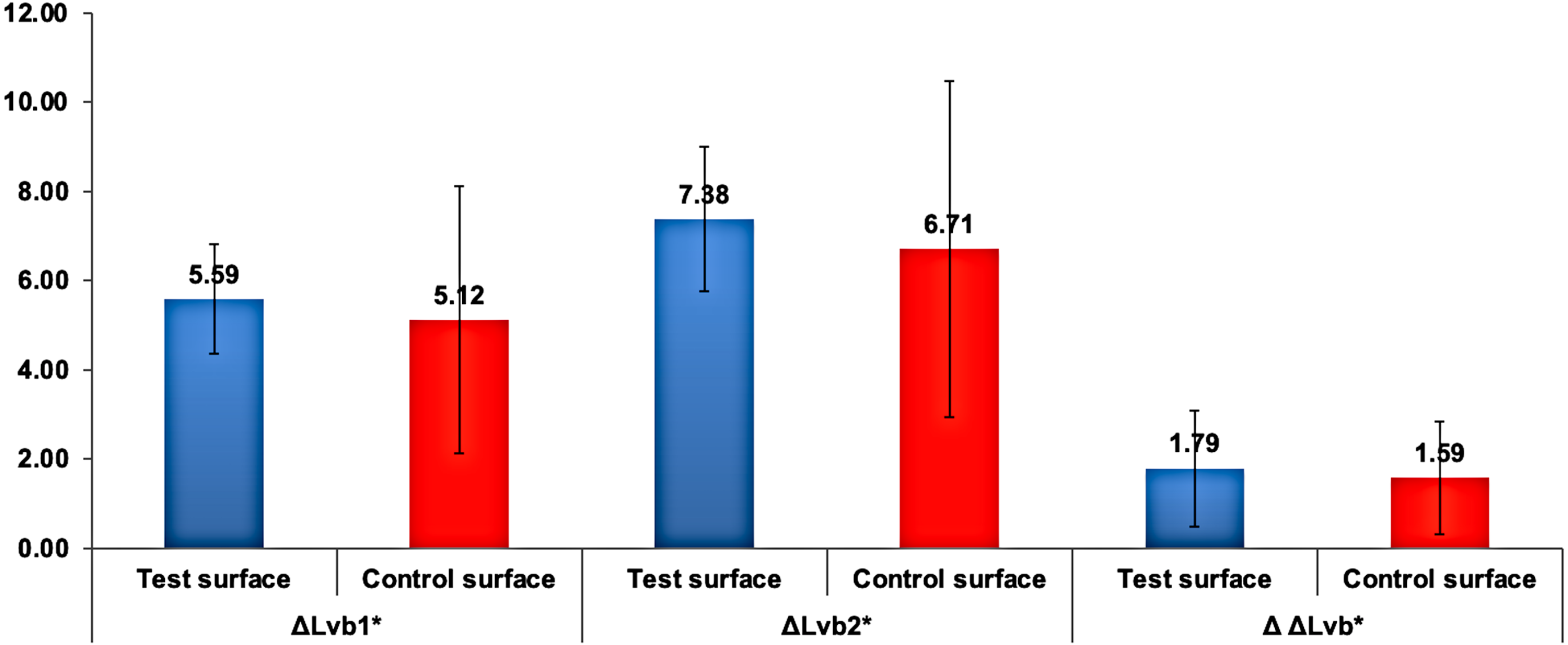
Mean change in Δ*L*_*vbx*_∗ and ΔΔ*L*_*vb*_∗ in test and control surfaces due to bleaching. $\Delta {L}_{vbx}^{\ast }$ is the change in lightness due to bleaching, where b indicates bleaching and x indicates the bleaching round number. ΔΔ*L*_*vb*_∗ is the change in lightness between bleaching rounds.

Multiple comparisons with Bonferroni revealed a statistically highly significant higher ${L}_{vb1}^{\ast }$ and ${L}_{vb2}^{\ast }$ than *L*_*v*__*staining*(8*days*)∗_ (*p* < 0.001). Mean ${L}_{vb1}^{\ast }$ was statistically highly significantly higher than mean ${L}_{vb2}^{\ast }$ (*p* < 0.001). However, the difference between ${L}_{vb1}^{\ast }$ and ${L}_{vb2}^{\ast }$ mean values was not statistically significantly different between the test and control surfaces (*p* = 0.4). The mean lightness ${L}_{vstaining(8)days}^{\ast }$) was used as a baseline to compare the change in lightness between the test and control surfaces. Independent samples *t*-test for comparing the change in the lightness ΔΔL${}_{vb}^{\mathrm{\ast }}$, ΔL_*vb*1_*, and ΔL${}_{vb2}^{\ast }$ between test and control surfaces revealed no statistically significant differences, as demonstrated in Fig. 6.

### Assessment of bleaching effect on capsule-coffee-stained enamel surfaces change of color

**Figure 7:**
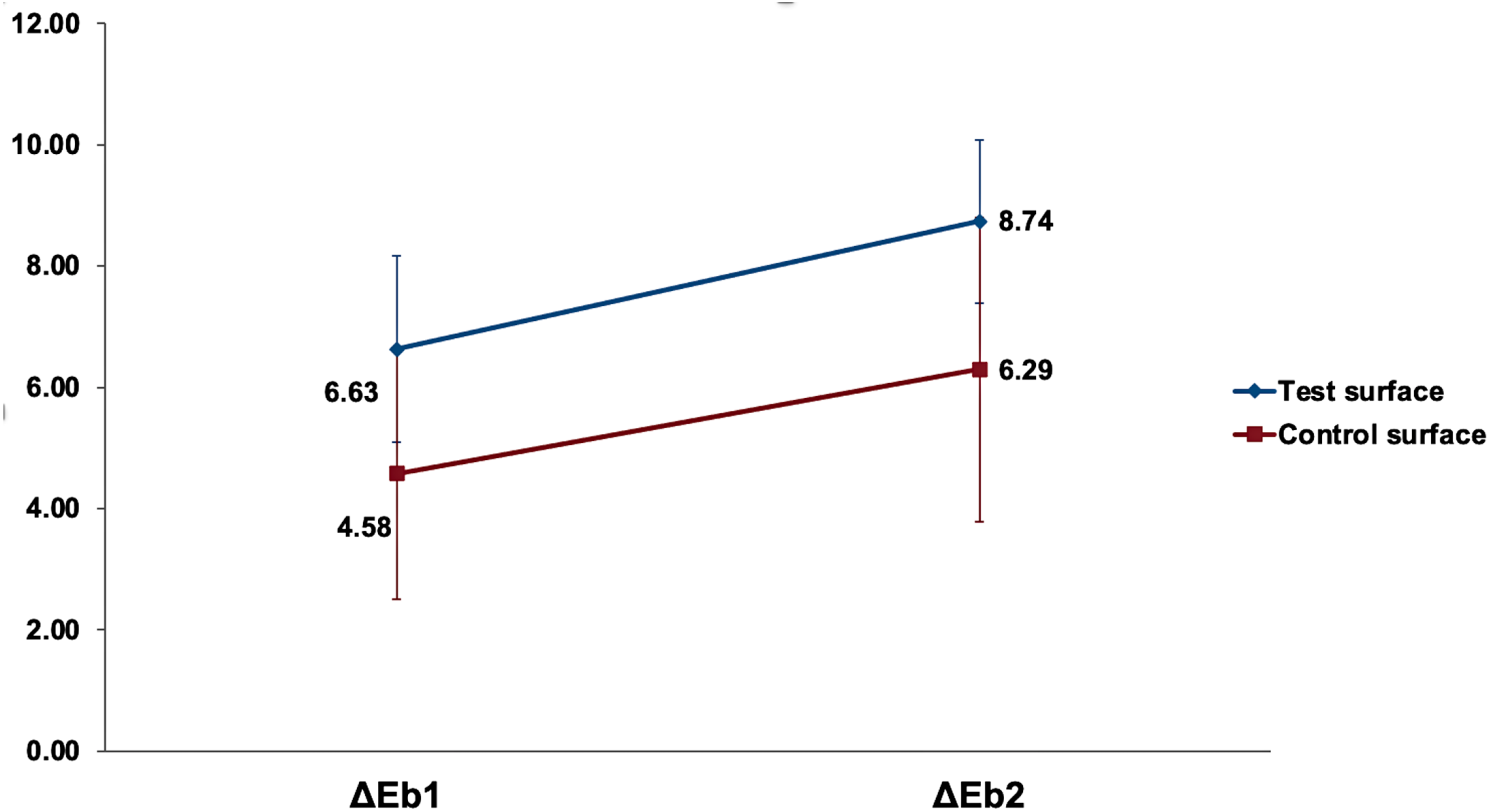
The increase in mean change of color (Δ*E*_*bx*_) of the test and control surfaces after the first and second bleaching rounds. Δ*E*_*bx*_ is the change of color from 8 days of staining and after bleaching, where b represents bleaching values, and x is the bleaching round.

Figure 7 shows the increase in change of color (ΔE_*bx*_) mean values of the test (received ICON infiltration treatment) and control (no ICON infiltration done) surfaces after the first and second bleaching rounds.

All variables were normally distributed, and parametric tests were used. General linear model repeated measure mixed design ANOVA was used to analyze the effect of bleaching rounds on the color change of test and control surfaces. Analysis of bleaching round as a main effect on the (ΔE_*b*_) values of surfaces was statistically highly significant (*p* <0.001). In contrast, the interaction of bleaching round with surfaces under investigation was non-significant (*p* = 0.49). However, the color change (ΔE_*b*_) when analyzing the type of surface as a main effect was statistically significantly higher in the test surfaces than in the control surfaces (*p* = 0.01). Pairwise comparison by Bonferroni between the first and second bleaching rounds revealed a highly significant increase in the change of color of tooth structure (*p* < 0.001). Pairwise comparison by Bonferroni revealed a statistically significant higher change of color in test surfaces compared to control surfaces (*p* = 0.01). Independent samples *t*-test revealed a statistically significantly higher (ΔΔE_*b*_) in test surfaces compared to control surfaces (*p* = 0.02), as detailed in Table 4.

**Table 4:** Independent *t*-test comparison of the change of color (ΔΔ*E*_*b*_) between test and control surfaces.

95% Confidence interval of the difference										
	ΔΔ*E*_*b*_	N	Mean	SD^1^	SEM^2^	Mean difference	SED^3^	Lower	Upper	*p*-value
Test	Δ*E*_*b*2_ − Δ*E*_*b*1_	10	6.33	1.53	0.48	2.05	0.82	0.34	3.76	0.021719^*^
Control		10	4.58	2.08	0.66					

**Notes.**

1 SD, standard deviation

2 SEM, standard error of mean L

3 SED, standard error of ΔΔ*E*_*b*_.

* statistically significant difference.

## Discussion

Several techniques and instruments are available to assess color changes of dental hard tissues and restorative materials in response to various therapeutics and local factors (Araujo et al., 2015; Lima et al., 2008). In the current study, spectrophotometry using a handheld device was chosen to objectively and quantitively measure the color parameters of test and control surfaces at several time points following their exposure to capsule-coffee staining solution (Dietschi, Benbachir & Krejci, 2010; Hardan et al., 2022).

One of the aims of this study was to assess the effect of intense exposure to popular dark capsule-coffee beverages on the color stability of dental hard tissues treated with the ICON^®^ resin infiltration system compared to non-infiltrated WSLs. Changes in color (ΔE_*x*_, where _x_ represents days of staining), which indicate the magnitude of color change between different time points (Paris et al., 2013), and changes in lightness (ΔL${}_{v}^{\ast }$) calculations were conducted using the ${L}_{\mathrm{v}}^{\ast }$ (tooth structure’s lightness distance in space color from the closest VITA classical shades A1-D4) and CIE L*, a*, and b* mean values after ICON resin infiltration of the test surfaces as the baseline values. The CIE L*, a*, and b* (Luo, 2014) values of test and control surfaces were recorded at baseline, 2 days, 4 days, 6 days, and 8 days storage in a capsule-coffee solution for the assessment of overall color changes (ΔE_x_). The CIE L*a*b* system is commonly used in the literature to objectively determine color-related property changes in both dental hard tissues and resins (Tan et al., 2015; Quek et al., 2018; Bahbishi et al., 2020). Using this standardized approach to assess color changes longitudinally allows comparison with previous studies, enabling better determination of changes in lightness and hues (Hammad, El-Wassefy & Alsayed, 2020; Bahbishi et al., 2020). However, it must be kept in mind that slight color changes are not perceivable by the human eye unless the ΔE values are equal to or larger than 3.7, beyond which esthetics would be of concern (Johnston & Kao, 1989).

In this study, extracted human teeth were used. Natural dentition shades and composition vary between individuals and different teeth in the oral cavity depending on several factors, including genetics and calcification variation (Kuckreja et al., 2017). To reduce the possibility of variation in color and calcification between test and control surfaces in the study, buccal and lingual surfaces of the same tooth were compared. Micro-computed tomography was used to confirm the formation of subsurface lesions that would be like clinically detected WSLs. This is a validated method for detecting subsurface lesions (Oliveira et al., 2020).

The staining and color changes of teeth and resin-based restorative materials, especially in the presence of the habitual consumption of certain substances, adversely affect esthetics and therefore influence the clinical lifespan of esthetic restorative treatment (Araujo et al., 2015; Tan et al., 2015). Resin-based materials containing tri ethylene glycol dimethacrylate (TEGDMA) monomer are hydrophilic, resulting in higher water absorption rates (Araujo et al., 2015; Ertas et al., 2006; Dos Santos et al., 2010; Fontes et al., 2009; Bagheri, Burrow & Tyas, 2005; Polak-Kowalska & Pels, 2019). Water is the vessel for moving staining pigments within the resin matrix. Therefore, high resin matrix to filler ratios and high water absorption rates would result in a high staining susceptibility (Hasanain, 2022; Bahbishi et al., 2020). TEGDMA resins have higher water absorption rates and stainability than other resins, such as Bis-GMA and UDMA (Araujo et al., 2015; Ertas et al., 2006; Dos Santos et al., 2010; Fontes et al., 2009; Bagheri, Burrow & Tyas, 2005; Polak-Kowalska & Pels, 2019). ICON^®^ resin infiltration materials contain TEGDMA. Thus, stains and dyes present in consumed beverages would be readily absorbed by the material (Paris & Meyer-Lueckel, 2010; Araujo et al., 2015). In 2019, Zhang, Li & Zhao (2019) compared the color change of ICON^®^ infiltrated enamel to blocks of other resin-based restorative materials and concluded its higher staining susceptibility (Zhang, Li & Zhao, 2019). In the current study, coffee was chosen to stain the specimens as suggested by Park et al. (2010) to produce perceivable color changes in both enamel and resin materials. Specifically, capsule-espresso-coffee was utilized due to its increased popularity as a daily consumed beverage worldwide (Lolli et al., 2020). Since the average coffee drinker consumes approximately 3.2 cups of coffee per day (minutes/cup almost 15 min) (Ertas et al., 2006), 24 h of storage in coffee would roughly represent one month of coffee drinking (Ertas et al., 2006; Gamal, Safwat & Abdou, 2022; Hasanain, 2022). Several studies on the effect of staining beverages on restorative materials and tooth structure have utilized this immersion model of staining (Gamal, Safwat & Abdou, 2022; Hasanain, 2022; Quek et al., 2018; Lel et al., 2016; Sarkis, 2012; Borges et al., 2011). In the current study, the samples were stored for eight days of 24 hours/day, representing approximately 8 months of coffee drinking.

Mean values of the color parameter ${L}_{vbaseline}^{\ast }$ and change of color (ΔE_*baseline*_) were not significantly different between test and control surfaces at baseline (*p* > 0.05). However, a significant color variation was detected following their exposure to the capsule-coffee solution. Analysis of time as a main effect on the (ΔE_*x*_) values of surfaces was statistically highly significant (*p* < 0.01), which indicated a significant increase in the color change of both test and control surfaces as exposure time increased. More specifically, the change in color between the second (ΔE_2_) and fourth (ΔE_4_) days compared to the eighth (ΔE_8_) days of exposure increased significantly (*p* = 0.03 and *p* = 0.01, respectively), indicating that significant color changes mainly occurred after the second day of intense coffee exposure. Thus, the null hypothesis that exposure time did not affect the color change of demineralized surfaces was rejected.

When analyzing the change in lightness ($\Delta {L}_{v}^{\ast }$) in relation to intense capsule-coffee exposure time, a statistically significant decrease in lightness was detected (*p* < 0.01), especially when comparing the eight (${L}_{vstaining(8days)}^{\ast }$) days of staining and the second (${L}_{vstaining(2days)}^{\ast }$) day of staining (*p* = 0.03). This showed that with increased exposure to capsule coffee, the demineralized dental surfaces darkened significantly regardless of treatment. This finding is supported by previous studies investigating the stainability of WSLs to coffee exposure (Araujo et al., 2015; Manno et al., 2018; Mori et al., 2016). On the other hand, the color stability of resin-infiltrated WSLs in the absence of coffee exposure was demonstrated by Aswani et al. (2019). Additionally, in a study by Liporoni et al. (2010), coffee did not produce a significant color change in un-infiltrated but bleached WSLs in relation to time after bleaching. In the current study, both the interaction of time with surfaces under investigation and the type of surface (test vs. control) as the main effect were non-significant (*p* = 0.47 and *p* = 0.35, respectively). This is comparable to the results of Lel et al. (2016) regarding the absence of a significant effect on color change when considering resin infiltration as the main effect and comparing two time points.

Mean values of color change (ΔE_*x*_) in response to intense capsule-coffee exposure were statistically significantly higher in resin-infiltrated surfaces (test) compared to untreated demineralized surfaces (*p* < 0.01). The significantly higher color change values in test (resin-infiltrated WSLs) surfaces were detected in all four exposure time points (*p* < 0.01). This would indicate that resin-infiltrated WSLs were more susceptible to staining due to capsule-coffee consumption than untreated WSL surfaces. A previous study also demonstrated the increased susceptibility of resin-infiltrated WSLs when teeth were stained using red wine (Lel et al., 2016). The lightness and change of lightness of the resin-infiltrated surfaces were lower than those of untreated demineralized surfaces; however, this was statistically insignificant (*p* = 0.35). This would indicate that the significant increase in the change in color (ΔE_*x*_) between resin-infiltrated and untreated WSLs was related to color hues, which were higher (yellower and redder) in test surfaces compared to control surfaces, rather than merely an increase in surface darkness. Therefore, we partially rejected the null hypothesis that no color change difference exists between infiltrated and non-infiltrated WSLs regardless of staining solution exposure time.

Bleaching is one of the most popular conservative esthetic treatment modalities for discolored tooth structure (Mourouzis, Koulaouzidou & Helvatjoglu-Antoniades, 2013; de Geus et al., 2016; Kihn, 2007). Its effectiveness is directly related to oxidizing agent concentration and duration of the bleaching procedure (Zekonis et al., 2003). In-office bleaching with high concentration hydrogen peroxide (35%) gel was used in the current study. The bleaching material was applied as recommended by the manufacturer, according to whom two to three bleaching rounds would produce favorable and lasting results. Literature suggests that more than one round of in-office bleaching may be required to reach the patient’s desired tooth color (de Silva Gottardi, Brackett & Haywood, 2006). In the current study, two rounds of bleaching sessions were performed on all samples, like in previous studies in the literature (Mourouzis, Koulaouzidou & Helvatjoglu-Antoniades, 2013; Hubbezoglu et al., 2008; Yami et al., 2020), and both the color change and lightness were significantly higher after the second round of bleaching (*p* < 0.001). The difference in change of color (ΔΔE_*b*_) after the second round of bleaching was significantly higher in infiltrated WSL surfaces than in untreated WSLs. However, the change of lightness was not significantly different between infiltrated and untreated WSLs regardless of the rounds of bleaching (*p* > 0.05). In 2006, a study concluded the possibility of producing a 2.1 to 3.7 shade difference with only one round of in-office bleaching (de Silva Gottardi, Brackett & Haywood, 2006). Hafez et al. (2010) reported minimal color change in composite resin materials with a single 45-minutes-long round of in-office bleaching using a 38% hydrogen peroxide gel. However, it was reported by other studies that a decrease in color change is to be expected 7–14 days after the first round of bleaching (Zekonis et al., 2003; Kutuk et al., 2018). ICON^®^ resin infiltration material is TEGDMA-based resin. Mourouzis, Koulaouzidou & Helvatjoglu-Antoniades (2013) found that the color of silorane-based resin materials is more susceptible to bleaching than other dimethacrylate-based composite resin materials.

In the current study, teeth samples were kept wet after each round of bleaching to avoid dehydration effects on the shade of dental structures (Kutuk et al., 2018) and to mimic the oral environment. The null hypothesis that there is no significant difference in the effect of bleaching between stained infiltrated and non-infiltrated WSLs was rejected because the results of the current study showed bleaching caused a significantly higher change in color (ΔE_*b*_) in the discolored infiltrated surfaces compared to untreated WSLs surfaces (*p* = 0.01). This suggests a higher susceptibility of resin-infiltrated WSLs to bleaching than untreated discolored WSLs. The mean change in lightness in both test (resin-infiltrated WSLs) and control (untreated WSLs) surfaces was around five after the first bleaching round and around seven after the second round of bleaching, which is considered clinically perceivable by most observers (Khashayar et al., 2014). The mean change in lightness ($\Delta {L}_{vb}^{\ast }$) of both discolored resin-infiltrated and untreated WSLs increased significantly after the first and second bleaching rounds (*p* < 0.01). This suggests the effectiveness of bleaching in esthetic management of discoloration of both infiltrated and untreated WSLs. Bleaching reversed the adverse color effects of staining on resin-infiltrated surfaces in a previous study by Jansen et al. (2021). This is in accordance with the results of Schoppmeier et al., where bleaching masked fluorosis effects on teeth in a manner that is similar to resin infiltration and even enhanced the esthetic outcome of resin-infiltration treatment of dental tooth structures (Schoppmeier et al., 2018). Bleaching with 16% carbamide peroxide was found to be effective in improving the color of stained resin infiltrated tooth structure (Araujo et al., 2015). However, in one study, using 10% carbamide peroxide bleaching did not produce an esthetic effect on resin-infiltrated WSLs. Still, in another study, home-bleaching did reverse coffee-induced staining effects on untreated WSLs (Bazzi et al., 2012; Santos et al., 2012). Additionally, a previous study in 2020 suggested the limited effectiveness of bleaching on resin-infiltrated WSLs afflicted with staining (Rocha et al., 2020). However, in the current study, a high concentration of hydrogen peroxide in-office bleaching gel was used instead of the 10% carbamide peroxide used in the 2020 study, which could explain the different results.

## Conclusions

Within the current study’s limitations, it can be concluded that resin infiltrated demineralized tooth structure is more susceptible to staining due to dark beverage consumption and to treatment with bleaching afterward. Thus, bleaching may be recommended for patients suffering from stained WSLs, even after treatment with resin infiltration. Increased exposure time to capsule coffee stains demineralized tooth structure even if the white spot lesions (WSL) were infiltrated with resin. Further research is, however, needed to further investigate the time-dependent shade stability of bleached WSLs and if further staining due to coffee consumption can, in fact, be reversed by additional bleaching cycles in resin-infiltrated WSLs.

##  Supplemental Information

10.7717/peerj.14135/supp-1 Supplemental Information 1 *L*_*v*_ means for all surfaces with staining time and statistical analysis of its values

10.7717/peerj.14135/supp-2 Supplemental Information 2 Statistical analysis of lightness and color change with bleaching

10.7717/peerj.14135/supp-3 Supplemental Information 3 delta E raw data for all surfaces during coffee immersion and after bleaching and L value after bleaching for all surfaces

## Additional Information and Declarations

### Data Availability

The following information was supplied regarding data availability: The raw data are available in the Supplementary File.
